# THUNDER 2: THeragnostic Utilities for Neoplastic DisEases of the Rectum by MRI guided radiotherapy

**DOI:** 10.1186/s12885-021-09158-9

**Published:** 2022-01-15

**Authors:** Giuditta Chiloiro, Davide Cusumano, Luca Boldrini, Angela Romano, Lorenzo Placidi, Matteo Nardini, Elisa Meldolesi, Brunella Barbaro, Claudio Coco, Antonio Crucitti, Roberto Persiani, Lucio Petruzziello, Riccardo Ricci, Lisa Salvatore, Luigi Sofo, Sergio Alfieri, Riccardo Manfredi, Vincenzo Valentini, Maria Antonietta Gambacorta

**Affiliations:** Fondazione Policlinico Universitario “A. Gemelli” IRCCS, Largo Agostino Gemelli 8, 00168 Rome, Italy

**Keywords:** Magnetic Resonance guided Radiation Therapy, Rectal cancer, Chemoradiotherapy, Early Regression Index, Radiomics

## Abstract

**Background:**

Neoadjuvant chemoradiation therapy (nCRT) is the standard treatment modality in locally advanced rectal cancer (LARC). Since response to radiotherapy (RT) is dose dependent in rectal cancer, dose escalation may lead to higher complete response rates. The possibility to predict patients who will achieve complete response (CR) is fundamental. Recently, an early tumour regression index (ERI) was introduced to predict pathological CR (pCR) after nCRT in LARC patients.

The primary endpoints will be the increase of CR rate and the evaluation of feasibility of delta radiomics-based predictive MRI guided Radiotherapy (MRgRT) model.

**Methods:**

Patients affected by LARC cT2-3, N0-2 or cT4 for anal sphincter involvement N0-2a, M0 without high risk features will be enrolled in the trial. Neoadjuvant CRT will be administered using MRgRT. The initial RT treatment will consist in delivering 55 Gy in 25 fractions on Gross Tumor Volume (GTV) plus the corresponding mesorectum and 45 Gy in 25 fractions on the drainage nodes. Chemotherapy with 5-fluoracil (5-FU) or oral capecitabine will be administered continuously.

A 0.35 Tesla MRI will be acquired at simulation and every day during MRgRT. At fraction 10, ERI will be calculated: if ERI will be inferior than 13.1, the patient will continue the original treatment; if ERI will be higher than 13.1 the treatment plan will be reoptimized, intensifying the dose to the residual tumor at the 11^th^ fraction to reach 60.1 Gy.

At the end of nCRT instrumental examinations are to be performed in order to restage patients. In case of stable disease or progression, the patient will undergo surgery. In case of major or complete clinical response, conservative approaches may be chosen. Patients will be followed up to evaluate toxicity and quality of life.

The number of cases to be enrolled will be 63: all the patients will be treated at Fondazione Policlinico Universitario A. Gemelli IRCCS in Rome.

**Discussion:**

This clinical trial investigates the impact of RT dose escalation in poor responder LARC patients identified using ERI, with the aim of increasing the probability of CR and consequently an organ preservation benefit in this group of patients.

**Trial registration:**

ClinicalTrials.gov Identifier: NCT04815694 (25/03/2021).

## Background

Neoadjuvant chemoradiation therapy (nCRT) currently represents the standard treatment modality in locally advanced rectal cancer (LARC) [1], achieving a pathological complete response (pCR) in approximately 11–42% of these patients, regardless of the initial disease stage [2–4].

Several studies have shown that patients achieving pCR usually have a better prognosis in terms of local control (LC), metastases-free survival (MFS) and overall survival (OS), representing a sound background for dose escalation approaches, aimed to enhance the therapeutic performance of nCRT [4–6]. Furthermore, conservative surgical approaches have recently been investigated in patients showing clinical complete response (cCR) after nCRT [7]. Both local excision (LE) and “Watch and Wait” (W&W) approaches represent to date, feasible options in order to reduce morbidities and toxicities related to unnecessary Total Mesorectal Excision (TME) in the case of successful nCRT [8, 9].

The possibility to predict the patients who will achieve complete response (CR) before surgery or even during nCRT is therefore of utmost importance, paving the way to the most innovative paradigms of a fully personalized medicine.

Several prediction models have been developed to predict CR in LARC patients, providing clinicians with valuable decisional support systems (DSS) for multidisciplinary oncological care personalization, so that patients predicted as “not responding” may take advantage of intensified treatments, while those predicted as “responding” may be addressed to more conservative therapeutic approaches, aiming to achieve organ preservation [10].

Amongst the different published models, a significant number focused on the possibility to predict pCR by analysing medical images, with the large majority of them focusing on Magnetic Resonance Imaging (MRI), as this imaging modality represents the gold standard technique for rectal cancer diagnosis and staging [11, 12]. Different MR based models revealed predictive value of the images acquired before [10, 13], during [14, 15], or after the end of CRT [16–18], supporting the possibility to identify imaging based biomarkers of response and modulate the treatment accordingly.

Fiorino and colleagues recently proposed an early regression index (ERI), which compares the Gross tumour volume (GTV) measurements at the time of simulation imaging acquisition and at the 10^th^ fraction, during the second week of treatment, as a potential biomarker for pCR prediction and more recently, also for long-term disease-free survival (DFS) [19, 20].

The original experience was performed on standard staging images acquired on 1.5 Tesla T2-weighted MRI and was later validated on an independent cohort of 52 LARC patients, who had undergone nCRT on a 0.35 T MRI hybrid MRI-LINAC, reporting an area under curve (AUC) of 0.93 in the case of an ERI_TCP_ value lower than 13.1 [15].

Since the response to radiotherapy is dose dependent in rectal cancer [21], patients with ERI > 13.1 values could theoretically benefit from more aggressive loco-regional treatments, justifying a dose escalation to the residual tumour by inducing a strong local immune reaction, helping in reducing also the risk of (or postponing) any metastatic spread [22–24].

The purpose of “THeragnostic Utilities for Neoplastic DisEases of the Rectum” (THUNDER 2) by MRI guided radiotherapy single centre prospective trial is to apply the ERI, in order to increase dose at 60.1 Gy on the residual primary tumour in the second week of treatment by MRI guided radiation therapy (MRgRT) in patients with a low prediction of pCR, treated with an MRI-LINAC hybrid machine.

Furthermore, such trial will allow to collect a relevant number of images and clinical data, that make it possible to investigate the possibility of improving the ERI_TCP_ performance, by integrating such indicator with delta radiomics features, which have already demonstrated promising predictive performance in rectal cancer [14, 25].

## Methods/design

### Study Design

**Fig. 1:**
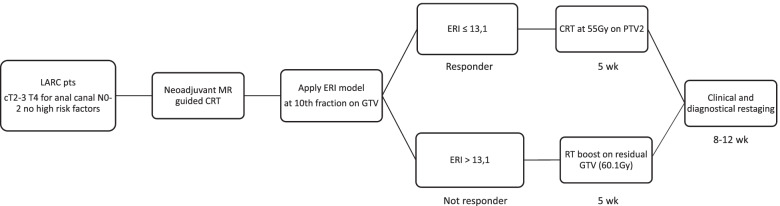
THUNDER 2: THeragnostic Utilities for Neoplastic DisEases of the Rectum by MRI guided radiotherapy treatment algorithm. LARC: locally advanced rectal cancer; CRT: chemoradiotherapy; GTV: gross tumor volume; ERI: early regression index; PTV: planning target volume; RT: radiation therapy; MR: magnetic resonance.

This is a single center prospective clinical trial. Fig. 1 describes the proposed treatment algorithm.

### Study objectives

The study will enroll patients affected by LARC and treated on a hybrid 0.35 T MR-LINAC (MRIdian, ViewRay Inc., Mountain View, CA, USA). The ERI will be calculated at the 10^th^ fraction of RT, in order to support the decision to deliver a RT dose boost on GTV.

The primary aim of this trial is to obtain the 10% increase of CR rate in the patients predicted as “not responders” (ERI > 13.1). Furthermore, the feasibility of delta radiomic-based predictive models in MRgRT will be assessed, in order to integrate the ERI model with omics information and improve its predictive performances [15].

Secondary objectives of the trial are to assess the 3 years LC, MFS, DFS, OS; the R0 resection rate; the tumor regression grade (TRG) 1 and TRG 2 rates [26]; the neoadjuvant rectal (NAR) score; the sphincter preservation rate; the organ preservation rate; the rectal and sexual functions.

### Ethics informed consent and safety

The final protocol was approved by the ethics committee of Fondazione Policlinico Universitario “A. Gemelli”, IRCCS of Rome, Italy (ethics committee identifier code 3460). This study is conducted in accordance with the most recent version of the Declaration of Helsinki and with the Italian laws and regulations. Any change to the protocol that may have an impact on the conduct of the study, the potential benefit to the patient, or that may affect patient safety, including changes in study objectives, study design, patient population, sample size, study procedures, or significant administrative aspects, will require a formal amendment to the protocol that will be approved by the Ethics Committee.

The protocol has been written according to the principles of good clinical practice (GCP).

Prior to inclusion in the trial, each patient will personally sign and date a written informed consent. The rationale, benefits and possible side effects will be explained in details to the patients before the voluntary signing of the informed consent. No intervention is carried out prior to the signing of the informed consent. Unexpected serious adverse events will be managed by the medical staff and recorded by the study data manager. All information relating to the study will be stored anonymously and securely.

### Statistics

According to our previous internal validation of the ERI_TCP_ model on MRIdian images of 43 patients, patients presenting an ERI > 13,1 that have a probability to obtain pCR is 3% [15]. Considering that the primary aim of the trial is to increase the CR rate in patients predicted as “responding” by the ERI to 13%, the calculation of the sample size was carried out using p0 = 3% and p1 = 13%. Using a confidence level of 95% and a power of 80%, a trial size of 42 cases was calculated with a cut-off of at least 4 recovered patients, to accept that a phase III trial should be undertaken [27, 28].

Considering that 84% of LARC patients undergoing nCRT do not achieve pCR, the total number of patients to be enrolled for the clinical protocol is 50 (42: 0.84). Moreover, considering a drop-out rate of 20%, the total number of patients to be enrolled is therefore 63. All the patients will be treated at Fondazione Policlinico Universitario A. Gemelli IRCCS in Rome.

Clinical patients data will be prospectively collected using BOA (Beyond Ontology Awareness), a software used for standardized clinical data collection for research purposes in our institution [29]. Patients data will be integrated in large data warehouse, property of Fondazione Policlinico Universitario Agostino Gemelli IRCCS of Rome.

MR images and contours will then be exported to the MODDICOM [30] advanced image analysis platform and radiomic features will be extracted and analyzed in terms of absolute values and delta ones (calculated as the ratio between the simulation value and the single fractions ones).

First order histogram, morphological, textural and fractal features will be analyzed first, following the methodology proposed, previously described by our group in Boldrini et al. [14, 25, 31].

The most significant features predicting CR will then be selected, using AUC and Mann–Whitney test.

Tumour clinical (i.e. cT, cN) and geometrical features (i.e. volume, surface, volume/surface ratio) will be finally added to setup a multivariate logistic model (based on a generalized linear model) to predict clinical and pathological CR.

Model performance will be evaluated by receiver operating characteristic (ROC) curve and internal bootstrapping for calibration errors detection (TRIPOD classification 1b) [32].

### Stratification

0.35 T MR images will be acquired at simulation and daily, throughout the whole MRgRT treatment and ERI will be calculated at fraction 10.

If ERI will be < 13.1, the patient will continue the prescribed treatment; otherwise, for ERI values > 13.1, treatment plan will be personalized and reoptimized considering the residual tumor at fraction 10 as a new target volume, where the dose will be intensified, reaching 60.1 Gy. The study workflow is described in Fig. 1.

### Patient selection

Besides general and patient’s specific criteria, a staging MRI of adequate technical quality is mandatory. The inclusion and exclusion criteria are presented in Table 1.

**Table 1:** Inclusion and exclusion criteria

Inclusion criteria	
General	ECOG 0–1
	Age over 18 years
	Written informed consent
	Adequate hematological function: -Granulocyte count > 1500/microl -Hemoglobin level > 10 g/dl -Platelet count > 100,000/microl -ALT/AST: 7–45 UI/L
Primary tumour characteristics	Histological proven adenocarcinoma of the rectum cT2-3, N0-2 or cT4 for anal sphincter involvement N0-2a, M0
	Tumour located between 0 and 15 cm above the anal verge
Exclusion criteria	
General	Contraindications for MR
	Pregnancy or lactating female patients
	Prior radiotherapy in pelvic region
	Psychological, familial, sociological or geographical condition potentially hampering compliance with the study protocol and follow-up schedule; those conditions should be discussed with the patient before registration in the trial
	No other malignancies in the previous history (except skin and initial cervical cancer)
	Important comorbidities: severe cardiac or coagulative disease, moderate or severe restrictive/obstructive lung deficit, severe cognitive impairment, moderate and severe renal and hepatic impairment
	Neoadjuvant chemotherapy
Primary tumour characteristics	Mesorectal fascia involvement for tumor
	Extramesorectal nodes involvement
	Extramural venous invasion (EMVI)
	Rectal mucinous adenocarcinoma histology

*ECOG* Eastern Cooperative Oncology Group; *ALT* Alanine aminotransferase; *AST* Aspartate aminotransferase; *MR* Magnetic Resonance

### Radiotherapy setting

Patients who meet the enrollment criteria will undergo pre-operative CRT treatment that will last 5 weeks.

Patients will be treated on a 0.35 T MRI-LINAC (MRIdian, ViewRay Inc) hybrid machine.

The Fluxboard device (FluxboardTM, MacroMedics, The Netherlands) will be used to immobilize patients in the supine position in a personalized and comfortable setting.

The initial 25-s (sec) acquisition will be carried out to verify the irradiation field. Subsequently, for contouring and planning purposes, a high definition 175 s-sequence will be obtained. After about 20 min, a simulation CT scan will be acquired to provide electronic densities for planning purposes with the same immobilization systems previously used. In the end, a co-registration of the CT images with the 175 s MR images will be performed [33].

The GTV will be contoured on the simulation MRI acquired at the beginning of treatment and on the MRI at 10^th^ fraction and the ERI_TCP_ will then be calculated. If ERI_TCP_ will be lower than 13.1, the patient will continue the original treatment with a total dose of 55 Gy on PTV2.

If ERI_TCP_ will be higher than 13.1, treatment plan will be reoptimized considering GTV3 (at 10^th^ fraction) as a further therapy volume and the dose will be intensified up to 60.1 Gy on PTV3, thanks to the online adaptive approach.

The CTV1 includes the primary rectal tumor, total mesorectum and selected lymphatic drainage stations, and/which will be delineated manually according to the guidelines proposed by Valentini et al. [34] by a radiation oncologist with specific expertise in the treatment of lower GI malignancies.

Planning target volume (PTV) 1 will correspond to the CTV1 + 0.5 cm in all directions.

The CTV2 includes the primary rectal tumour plus the corresponding mesorectum. PTV2 is the CTV2 + 0.5 cm in all directions [33].

In case of the need for treatment intensification, the boost volume (PTV3) will be defined as the GTV delineated on MRI acquired at the fraction 10 (at 22 Gy) with an added isotropic margin of 0.3 cm.

The normal tissue volumes (i.e. organs at risk – OARs) to be contoured are bladder, small bowel, sigmoid colon, anal canal, femoral heads and iliac bones.

All the patients will start a long-course radiotherapy treatment consisting of 25 fractions, with a total prescription dose of 55 Gy in fractions of 2.2 Gy to PTV2 and 45 Gy in fractions of 1.8 Gy to PTV1, following a simultaneous integrated boost (SIB) protocol [33].

ERI_TCP_ will be calculated at fraction 10: a treatment intensification protocol will be administered in patients showing ERI_TCP_ > 13.1, while patients showing ERI_TCP_ ≤ 13.1 will continue the original treatment.

**Fig. 2:**
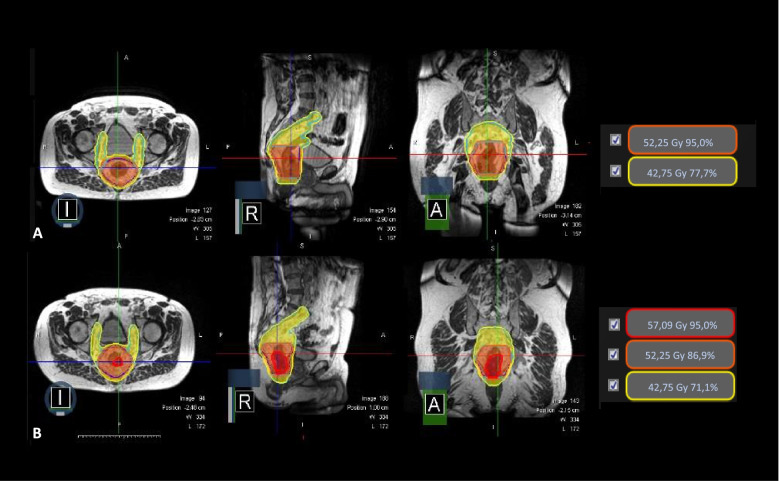
Example of dose escalation in accordance with the ERI index. Figure 2A shows the simulation plan according to the SIB 2 protocol. Figure 2B represents the dose escalation obtained at the tenth treatment fraction, where the red colourwash isodose line represents the V95% of the 60.1 Gy prescribed to PTV3. The orange colourwash isodose line represents the V95% of 55 Gy prescribed to PTV2, while the yellow colourwash isodose line represents the V95% of 45 Gy prescribed to PTV1

Treatment intensification will consist in a local dose boost to PTV3, where a fractional dose of 2.54 Gy will be delivered from fraction 11 to the end of treatment. PTV1 and PTV2 will continue the original prescription dose. At the end of the intensified treatment, PTV3 will have received a total physical dose of 60.1 Gy, which corresponds to 74.6 Gy in biologically effective dose (BED) and 62.2 in equivalent dose, assuming an alpha/beta equal to 10 [35]. Fig. 2 shows an example of dose escalation.

An “inverse planning” approach using computerized optimization will be used (Intensity Modulated Radiation Therapy – IMRT) and the prescription dose will be normalized to the target mean according to ICRU 83 [36].

The reported doses for each PTV will include the prescription dose, the maximum point dose, the % target volume receiving > 105% and > 110% of its prescribed dose, the % target volume receiving ≥ 95% of the prescribed dose, and the mean dose to the PTV’s. Doses to the OARs will also be recorded.

Concomitant chemotherapy (CHT) with 5-FU (225 mg/mq/day in continuous infusion) or oral capecitabine (1650 mg/mq/day chronomodulated) will be administered with no scheduled interruptions.

The patient will be positioned at the isocenter prior to each daily fraction. To improve the reproducibility, an internal protocol will be used to achieve stable conditions of bladder filling: patients will be instructed to drink 500 cc of water 30 min before simulation and before each treatment session.

In the case of dose escalation up to 60.1 Gy, the GTV3 will be recontoured and the plan reoptimized daily, while the patient is on the treatment couch, using an adaptive online RT approach in order to ensure target coverage and OARs sparing [37].

A cine-MRI gating protocol will be performed on PTV2 or PTV3, setting a 5% region of interest (ROI) value in a 3 mm boundary from the CTV2 or CTV3, therefore ensuring that the target volumes are always in the expected position. Delay time value is set at 0 s, so that the movement of the ROI outside the boundary will immediately trigger the beam off.

### Response and resectability evaluation

The clinical response will be evaluated with pelvis MRI and body CT or ^18^F-FDG PET-CT, repeated 8–10 weeks after the end of nCRT. Surgery which will consist of total or partial mesorectal excision, which will be performed 10–12 weeks after the end of CRT in case of partial, stable, or progression disease.

In case of major or complete clinical response at restaging imaging, endoscopic examination should be performed. In case of cCR, a watch and wait (W&W) or local excision (LE) approach could be followed according to the multidisciplinary tumor board (MDT) decision. The administration of adjuvant CHT will be discussed in the framework of MDT, based on clinical and pathological high-risk features.

The follow up surveillance will be scheduled depending on the chosen conservative or surgical approach as reported in Table 2 and 3, respectively. Clinical, instrumental, toxicity, and quality of life (QoL) assessment [38–40] will be performed during follow-up.

**Table 2:** Follow-up after neoadjuvant treatment in case of watch and wait (W&W) or local excision (LE)

	**Follow up time (months)**									
	**3**	**6**	**9**	**12**	**15**	**18**	**21**	**24**	**30**	**36**
Medical history, blood sample and DRE	x	x	x	x	x	x	x	x	x	x
Rectoscopy	x	x	x	x	x	x	x	x	x	x
MRI		x		x		x		x		x
Total body CT				x				x		x
Colonoscopy				x						x
Abdomen US	x		x		x		x		x	
QoL questionnaries	x			x				x		
Acute toxicity Grading (CTCAE v. 4.0)	x									
Late toxicity Grading (CTCAE v. 4.0)		x	x	x	x	x	x	x	x	x

Blood sample includes blood count with differential leukocyte count and CEA. The quality of life (QoL) questionnaires assess sexual (FSFI—Female sexual function index—and IIEF—International Index of Erectile Function—questionnaires) and bowel function (MSKCC BFI-Memorial Sloan-Kettering Cancer Center Bowel Function Instrument). *DRE* Digital rectal examination; *MRI* Magnetic resonance imaging; *CT* Computed tomography; *US* Ultrasound; *CTCAE* Common terminology criteria for adverse events

**Table 3:** Follow-up after neoadjuvant treatment in case of total mesorectal excision (TME)

	**Follow up time (months)**								
	**1.5**	**3**	**6**	**9**	**12**	**18**	**24**	**30**	**36**
Medical history, blood sample and DRE	x	x	x	x	x	x	x	x	x
Surgical histology	x								
Total body CT		x		x		x		x	
Abdomen US			x		x		x		x
Colonoscopy				x					x
QoL questionnaries		x			x		x		
Acute toxicity Grading (CTCAE v. 4.0)		x							
Late toxicity Grading (CTCAE v. 4.0)			x	x	x	x	x	x	x

Blood sample includes blood count with differential leukocyte count and CEA. The quality of life (QoL) questionnaires assess sexual (FSFI—Female sexual function index—and IIEF—International Index of Erectile Function—questionnaires) and bowel function (MSKCC BFI-Memorial Sloan-Kettering Cancer Center Bowel Function Instrument). *DRE* Digital rectal examination; *CT* Computed tomography; *US* Ultrasound; *CTCAE* Common terminology criteria for adverse events

## Discussion

One of the main goals of nCRT treatment for LARC patients is to achieve CR, which corresponds to improved survival outcomes [41]. Many preoperative strategies are reported in literature with the aim of increasing the CR rate. Studies on intensification of preoperative CHT have been conducted showing no particular benefit, except in the German CAO/ARO/AIO-04 study, where a DFS benefit was demonstrated with the addition of oxaliplatin to neoadjuvant and adjuvant CHT regimens [42]. Based on the assumption that the response of tumor also occurs after the end of RT treatment, it has also been showed that the rate of pCR is correlated with the prolongation of the interval between nCRT and surgery [5]. Furthermore, RT dose escalation correlates with the CR rate after nCRT. Appelt et al. demonstrated a significant correlation between dose and tumour regression when doses in the range of 50.4 to 70 Gy are achieved [41].

Radiotherapy doses ≥ 60 Gy correlate with a pCR rate of 20.4% and low rates of acute G3 toxicity (10.3%), consisting mostly of gastro-intestinal complaints, dermatitis, leukopenia/neutropenia, pain, and high rates of resectability (89.5%) in particular, are reported in the literature [21]. The increase in toxicity does not depend on an increase in dose, although it is expected that more advanced RT techniques may contribute to increased tolerability to treatment, due to a higher dose conformation that allows a more efficient OAR sparing [33, 43, 44]. The choice of achieving a dose of 60.1 Gy on the GTV was made according to the reported evidence in the literature and the dosimetric possibility of performing a SIB3 approach. Dose escalation starting from the eleventh treatment fraction with a prescribed dose per fraction of 2.54 Gy on the GTV was found to be satisfactory from a dosimetric point of view, in order to maintain adequate coverage and dose fall-off between the three PTVs obtained.

Different technologies may be applied in order to boost the dose on macroscopic disease, such as contact therapy (CRX) used for distal, small lesions in well-selected patients, enhancing sphincter preservation rates or the addition of brachytherapy boost to nCRT that leads to an increase in pCR, even if not to a corresponding increase in late outcomes [45].

In the framework of personalized treatments, it is necessary to exploit the benefits of available technologies to safely deliver the RT dose while sparing OARs.

MRgRT offers the opportunity to take advantage of MR imaging combined with innovative gating solutions. Furthermore, it is possible to optimize the treatment plan online on a daily basis, taking into account all the possible anatomical modifications, while the patient is still on the treatment couch [33, 37]. This may have a significant impact especially from the perspective of dose escalation, thanks to an efficient management of the different degrees of filling and position of the OARs surrounding the targets volumes (i.e. –bowel, bladder). Daily MR imaging also allows the response to treatment to be monitored and toxicities to be early intercepted/detected and even potentially avoided [46]. Considering the available therapeutic opportunities, it is therefore crucial to stratify treatment options based on disease and patient characteristics.

Indeed, LARC patients should be divided into two categories, which are the intermediate- and high-risk patients, based on the presence of extramural vascular invasion, involvement of mesorectal fascia, massive nodal involvement or enlarged lateral lymph nodes [47, 48].

These features suggest a high potential risk of developing distant metastases and systemic disease control becomes the priority, requiring systemic therapy intensification as demonstrated by the recent results of the RAPIDO trial [49]*.* Furthermore, results from the recent Prodige 23 trial, which enrolled patients with cT3 and cT4M0 tumors even with high-risk features, confirmed this evidence. The authors proposed an approach based on the inclusion of early chemotherapy in the treatment workflow (total neoadjuvant therapy—TNT) that has been shown to significantly improve 3y-DFS [50].

In contrast, for the group of intermediate-risk patients, LC is the key treatment endpoint. A predictive model able to detect patients who will achieve complete response versus those who are bad responders, may be of significant help to design a trial of RT dose escalation in this subset of patients.

In recent studies, researchers focused on changes in tumor volume on MR images acquired during treatment [51], observing in particular that the ERI index based on disease volumes on simulation and in the second week of treatment MR images, [20] correlates with pCR after nCRT for rectal cancer with high sensitivity and negative predictive value. This index was also validated in a following external cohort of patients using low tesla MR images, reporting promising results also in other tumor sites [15, 52].

To our knowledge, this is the first trial where an image based predictive model is used in clinical workflow to support the decision for a RT intensification on residual disease volume.

New exploratory studies are currently on-going to explore the potentialities of integrating ERI index with delta radiomic features: one of the main limitations of this indicator is in fact that it does not take into consideration the textural characteristics within the tumour, but only volumetric information. It is reasonable to suppose that the integration of radiomic features, describing the variation of heterogeneity within the tumor during the treatment, can effectively integrate the volumetric information, improving the predictive performance, as demonstrated in some recent reviews dealing this topic [53, 54].

The THUNDER 2 trial is designed to increase LC rates in the arm of patients with poor prognosis by applying the ERI index, thereby offering an innovative perspective on the management of LARC patients undergoing MRgRT. The aim of this study is therefore to further evaluate prospectively the predictive power of the ERI index alone and when combined with the analysis of delta radiomics features extracted from MR images during the course of RT treatment, to assess the "strength" of the two different models alone and in combination in a fully personalized and translational framework.

## Data Availability

The datasets used analysed during the current study are available from the corresponding author on reasonable request.

## Abbreviations

5-FU: 5- Fluorouracil
ADC: Apparent diffusion coefficient
ALT: Alanine aminotransferase
AST: Aspartate aminotransferase
AUC: Area under curve
BOA: Beyond ontology awareness
cCR: Clinical complete response
CHT: Chemotherapy
CR: Complete response
CRT: Chemoradiation therapy
CT: Computed tomography
CTCAE: Common terminology criteria for adverse events
CTV: Clinical target volume
DFS: Disease free survival
DRE: Digital rectal examination
DSS: Decisional support system
DWI: Diffusion-weighted imaging
EMVI: Extramural venous invasion
ERI: Early regression index
ERITCP: Tumor control probability based early regression index
FSFI: Female sexual function index
GTV: Gross tumor volume
ICRU: International commission on radiation units and measurements
IIEF: International Index of Erectile Function
IMRT: Intensity modulated radiation therapy
KBO: Knowledge-based oncology
LARC: Locally andvanced rectal cancer
LC: Local control
LE: Local excision
LINAC: Linear accelerator
MDT: Multidisciplinary tumor board
MFS: Metastases-free survival
MR: Magnetic resonance
MRgRT: Magnetic resonance guided radiation therapy
MRI: Magnetic resonance imaging
MSKCC BFI: Memorial Sloan-Kettering Cancer Center Bowel Function Instrument
NAR: Neoadjuvant rectal
nCRT: Neoadjuvant chemoradiation therapy
OARs: Organs at risk
OS: Overall survival
pCR: Pathological complete response
PTV: Planning target volume
QoL: Quality of life
ROC: Receiver operating characteristic
ROI: Region of interest
RT: Radiation therapy
SIB: Simultaneous integrated boost
SUV: Standardized uptake value
TRG: Tumor regression grade
US: Ultrasound
W&W: Watch and wait
